# Editorial: Cell Transplantation in Diabetes: Cell-Based Immunotherapy for Type 1 Diabetes

**DOI:** 10.3389/fendo.2022.887858

**Published:** 2022-05-02

**Authors:** Lingling Wei, Kazuhiko Yamada, Ping Wang

**Affiliations:** ^1^ Center for Endocrine Metabolism and Immune Diseases, Beijing Luhe Hospital, Capital Medical University, Beijing, China; ^2^ Beijing Key Laboratory of Diabetes Research and Care, Beijing, China; ^3^ Columbia Center for Translational Immunology, Department of Medicine, Columbia University Irving Medical Center, New York, NY, United States; ^4^ Department of Surgery, Columbia University Irving Medical Center, New York, NY, United States; ^5^ Precision Health Program, Michigan State University, East Lansing, MI, United States; ^6^ Department of Radiology, College of Human Medicine, Michigan State University, East Lansing, MI, United States

**Keywords:** type 1 diabetes mellitus, cell based therapy, immunotherapy, cell transplantation, autoimmune diseases

The Research Topic Cell Transplantation in Diabetes: Cell-Based Immunotherapy for Type 1 Diabetes, represents a collection of review articles, mini review articles and original research articles, which together describe the research status, latest strategies and development direction of islet transplantation and stem cells in type 1 diabetes (T1D) treatment.

Type 1 diabetes mellitus (T1D) is an autoimmune disease characterized by absolute insulin deficiency caused by pancreatic β-cell-specific immune damage (1). Cell-based immunotherapy aims to protect or even rebuild the endogenous insulin secretion system through pancreatic β-cell replacement or regeneration therapy which improves the disease process and prognosis (2). It is a research direction that has attracted much attention in the field of T1D treatment.

Islet transplantation has become an effective method for the radical treatment of advanced diabetes due to the advancement of isolation technology and transplantation protocols. However, there is a severe shortage of pancreatic donors suitable for islet transplantation, which limits its wide clinical application. Porcine islet xenotransplantation is a promising approach to overcome the bottleneck of islet therapy in T1D. However, the delayed insulin secretion caused by the immaturity and immunogenicity of neonatal porcine islets remains a challenge for its clinical application. In the field of islet transplantation, numerous studies have shown that mesenchymal stem cells (MSCs) are used to improve islet function and graft survival after transplantation. The mini review article by Koehler et al. reviewed the immunomodulatory and anti-inflammatory properties of MSCs in islet transplantation. Some challenges currently faced by islet xenotransplantation are also presented. Neonatal or young pigs could provide higher islet yield than adult pigs. The main disadvantage of porcine pancreatic islet cell clusters (ICCs) (neonatal or juvenile porcine islet cell populations) was the lack of integrity and maturity. Porcine MSCs could improve islet function of ICCs and graft survival after transplantation. Microencapsulation technology could address the need for immunosuppression and protect islets from immune attack while still enabling the exchange of oxygen, insulin and nutrients. However, delayed and impaired transplantation prognosis and the formation of encapsulated fibrosis due to immature islet cells still severely limited the clinical application of encapsulated islet transplantation.

When the islets were separated from the pancreatic tissue, the blood supply of the islets was destroyed. At that time and at the initial stage of transplantation, the islets only relied on the diffusion of oxygen in the surrounding environment to maintain their survival. Therefore, the oxygen partial pressure in the center of the islet was extremely low, resulting in the occurrence of cell death and apoptosis in the center of the islet. The revascularization of islets after transplantation was slow, and even after revascularization of the transplanted islets, their blood supply and partial pressure of oxygen were still significantly lower than those in normal pancreas. Therefore, accelerating the angiogenesis around islets after transplantation and increasing the density of blood vessels through corresponding strategies have become the focus of recent research in the field of islet transplantation. The mini review article by Pomposelli et al. introduced a new strategy for composite islet-kidney allo-transplantation for the simultaneous treatment of T1D and diabetic nephropathy. At present, most of the reports chose pancreatic islets to be transplanted into the liver through the portal vein. This site was rich in blood supply, and insulin secretion and release from the portal vein system were more in line with the physiological pathway of insulin, but it may cause serious complications such as portal hypertension and liver failure. Combined islet-kidney transplantation allowed islets to be transplanted under the renal capsule in advance, allowing them to be prevascularized in the host environment. This not only avoided instant blood-mediated inflammatory reaction (IBMIR) in the early stages of transplantation, but also provided a persistent physical barrier to the host’s innate immune response. In addition, a new strategy was to incubate islets with anti-inflammatory nanoparticles to reduce islet cell apoptosis after transplantation, which could reduce the number of donors required. It also solved the defect that only a limited volume of islet grafts can be accommodated under the renal capsule. By using a durable chimera strategy to induce tolerance in the host, transplant rejection can be avoided, thereby avoiding the long-term use of immunosuppressive drugs, and avoiding the side effects of immunosuppressive drugs. Moreover, placing the islet graft under the renal capsule made subsequent harvesting of the graft relatively easy, facilitating observation and exploration. Animal experiments of islet-kidney combined transplantation suggested that this strategy was an effective means for the treatment of T1D and end-stage renal disease. In order to further improve the clinical effect of this scheme, especially the function of transplanted islets, it needed to be used in combination with other strategies. Moreover, islet-kidney combined transplantation was still in the exploratory stage of animal experiments, and the long-term efficacy of clinical application needed to be further evaluated.

In addition to the limitation of donor organ shortage, islet transplantation therapy is also affected by autoimmune and allogeneic rejection as well as non-immune-related factors. Non-invasive techniques for monitoring and assessing islet graft fate *in vivo* are important for understanding the underlying causes of graft failure, reflecting the quality of successfully transplanted islets, as well as islet survival and insulin secretion *in vivo*. A review by Arifin and Bulte described how imaging techniques can be used to trace and monitor islet graft distribution, number or volume, viability, and function *in vivo*. They outlined bioluminescence (BLI), fluorescence microscopy imaging (FMI), Single-photon emission computed tomography (SPECT), Positron emission tomography (PET), magnetic resonance imaging (MRI), magnetic particle imaging (MPI) and ultrasound imaging, the advantages, limitations and clinical utility of each specific imaging method.

Currently, BLI and FMI had been widely used in the evaluation of transplanted pancreatic islets in animal models, but they were not suitable for clinical patient studies due to their invasiveness or lack of light penetration in deep tissues. PET, SPECT, MRI, MPI, and ultrasound were currently being developed for this specific purpose, and unlike BLI or FMI, they had great potential for clinical translation. For pancreatic islet imaging technology to be successfully applied in clinic, the labeling technology needed to achieve low toxicity of the marker, long and stable labeling time, and high detection sensitivity. Recent progress had been made in the field of *in vivo* islet imaging, and much work remained to be done to further improve the accuracy and efficacy of image-guided islet therapy.

Diabetes mellitus induced by immune checkpoint inhibitor (ICI) therapy is a rare but potentially life-threatening complication. It is unclear if ICI treatment causes selective islet toxicity or non-selective pancreas toxicity, for the purpose of answering this question, Zhang et al. conducted a retrospective cohort study of 11 patients with immune checkpoint inhibitor-induced type 1 diabetes (ICIT1D) in their original research article. In the study, they found that among patients with autoimmune diabetes, 25% (2/8) had elevated lipase levels and no patients (0/6) had elevated amylase levels. Combined with longitudinal clinical studies, the results suggested that ICI can induce selective pancreatic endocrine or exocrine toxicity. Studies suggested that GAD65 antibody was the most sensitive autoantibody associated with ICIT1D, but should not be used as a necessary criterion for diagnosis. The study also described various clinical manifestations of ICIT1D, with late onset age, rapid and significant pancreatic islet failure, and concurrent of other gland damages. Therefore, improving the understanding and attention of domestic doctors and patients to ICIT1D, early identification and timely initiation of insulin therapy may avoid serious complications of autoimmune diabetes, such as diabetic ketoacidosis (DKA).

Studies using preclinical animal models have demonstrated that transplantation of stem cell derived beta-like cells (sBCs) can rescue diabetes. However, the safety and efficacy of sBCs for clinical diabetes treatment and how sBCs respond to the inflammatory milieu of diabetic T cells in a rigorous human *in vivo* setting have not been systematically studied and demonstrated. It is well known that human β cells express human leukocyte antigen class I (HLA class I), and HLA class I is involved in the pathogenesis of T1D by expressing diabetogenic antigens to CD8 T cells. To study the interaction of human sBCs and T cells, a reproducible, well-defined *in vitro* assay system was established to measure the stimulation of sBCs by human autologous CD8 T cells in the original research article by Castro-Gutierrez et al. Specifically, the surface expression of HLA class I of sBC was knocked out using genome engineering, and the inducible overexpression of the immune checkpoint inhibitor programmed death ligand 1 (PD-L1) was integrated. The results showed that sBC stimulated human diabetic CD8 T cells in an HLA-dependent manner, and PD-L1 overexpression could effectively reduce this stimulation. This suggested that manipulation of the HLA class I and PD-L1 receptor could provide protection in humans against diabetes-specific immune recognition.

In conclusion, the studies outlined in this Research Topic demonstrate that cell therapy focuses on two core mechanisms of T1D development and exerts therapeutic effects on islet β-like cell replacement or immunomodulation. Islet transplantation directly achieves islet function reconstruction at the cellular level. Pluripotent stem cells provide an important source of cells for islet transplantation by inducing differentiation into islet β-like cells. MSCs can provide a favorable internal environment for the survival of β-like cells by inducing immune tolerance and immune regulation. The combination of different types of cell therapies may complement each other in terms of mechanisms, thereby improving efficacy, curbing or even reversing disease progression.

## Author Contributions

All the authors have made a substantial, direct and intellectual contribution to the work and approved it for publication.

## Conflict of Interest

The authors declare that this editorial was prepared in the absence of any commercial or financial relationships that could be construed as a potential conflict of interest.

## Publisher’s Note

All claims expressed in this article are solely those of the authors and do not necessarily represent those of their affiliated organizations, or those of the publisher, the editors and the reviewers. Any product that may be evaluated in this article, or claim that may be made by its manufacturer, is not guaranteed or endorsed by the publisher.
